# Classification of hyper-scale multimodal imaging datasets

**DOI:** 10.1371/journal.pdig.0000191

**Published:** 2023-12-13

**Authors:** Craig Macfadyen, Ajay Duraiswamy, David Harris-Birtill

**Affiliations:** University of St Andrews, St Andrews, United Kingdom; Harvard University, UNITED STATES

## Abstract

Algorithms that classify hyper-scale multi-modal datasets, comprising of millions of images, into constituent modality types can help researchers quickly retrieve and classify diagnostic imaging data, accelerating clinical outcomes. This research aims to demonstrate that a deep neural network that is trained on a hyper-scale dataset (4.5 million images) composed of heterogeneous multi-modal data can be used to obtain significant modality classification accuracy (96%). By combining 102 medical imaging datasets, a dataset of 4.5 million images was created. A ResNet-50, ResNet-18, and VGG16 were trained to classify these images by the imaging modality used to capture them (Computed Tomography (CT), Magnetic Resonance Imaging (MRI), Positron Emission Tomography (PET), and X-ray) across many body locations. The classification accuracy of the models was then tested on unseen data. The best performing model achieved classification accuracy of 96% on unseen data, which is on-par, or exceeds the accuracy of more complex implementations using EfficientNets or Vision Transformers (ViTs). The model achieved a balanced accuracy of 86%. This research shows it is possible to train Deep Learning (DL) Convolutional Neural Networks (CNNs) with hyper-scale multimodal datasets, composed of millions of images. Such models can find use in real-world applications with volumes of image data in the hyper-scale range, such as medical imaging repositories, or national healthcare institutions. Further research can expand this classification capability to include 3D-scans.

## Author summary

We are seeing an explosion in the volumes of diagnostic imaging data being acquired and stored digitally. Initial diagnoses of multiple diseases, including cancer, are often made on the basis of multiple modality scans of the same anatomical area, contributing to this proliferation of imaging data. When mixed modalities are stored in a large repository/data lake, classification of images into constituent modalities is a challenge, especially when involving millions of images. ML researchers collaborating with clinicians to build AI-based decision support systems could benefit from automatic modality classification, to help with image retrieval, archival, data balancing, and as a diagnosis-aid. Further, a classifier that operates based on a visual classification model, as opposed to label-based classification, can offset the time, cost, and errors involved in manual labelling and identification processes. In our paper, we identify a Deep Neural Network algorithm that can accurately perform classification on a mixed-modality hyperscale dataset (4.5 million images), with significant accuracy—the best of our models achieves 96% accuracy in classifying CT, MR, and PET modalities. These performance figures demonstrate classification capabilities that exceed those of more complex classifier implementations, especially at hyperscale imaging data volumes.

## 1. Introduction

With the proliferation of deep neural networks trained on heterogenous multimodal data to detect and predict diseases, there has been an explosion in the volume of diagnostic medical imaging data [1]. Clinicians often order multiple scans of the same patient in different modalities to gather evidence to make an improved diagnosis/prognosis [2]. Algorithms that can accurately classify a large heterogeneous dataset into its constituent modalities can be beneficial to researchers and clinicians, allowing them to automatically segment a particular type of modality for retrieval, archival, data balancing, and diagnostic purposes. Manual methods for classifying medical images are typically error-prone unless done by costly domain experts [3].

This paper outlines a deep neural network that accurately classifies a hyper-scale (4.5 million images), mixed-modality dataset into constituent modalities. The developed approach has significant benefit potential for researchers, clinicians, and imaging archives by helping effectively and efficiently classify diagnostic imaging data, in the magnitude of real-world volumes. While classification of hyperscale datasets have been attempted in other areas, such as Earth-science [4], including studies of plankton and marine snow [5], the proposed approach is novel in the field of classification of medical imaging modalities. This study aims to stimulate other hyper-scale projects in this area.

Multiple open-access data sets were used to build the hyper-scale multimodal dataset of 4.5 million images from sources such as The Cancer Imaging Archive [16], Stanford ML Group [17] the largest of which contains 262,000 chest X-ray images, and Kaggle [18] host labelled datasets.

The models trained on this hyper-scale multimodal dataset were a ResNet-18, ResNet-50 and a VGG16. When these models were tested for classification accuracy, the results are in the high 90%’s across the train, validate and test sets which shows that the models are able to classify with significant accuracy. The best performing model in this study, a ResNet18, achieves significant classification performance (96%+) on classifying CT, MR and PET modalities.

### 1.1. Previous literature

A number of research articles focus on deep learning systems to classify modalities in diagnostic imaging data. However, to the best of our knowledge, there have not been any examples of a system that combines medical imaging datasets at the hyper-scale (millions of images) level to perform modality classification.

Approaches to classifying medical imaging data by modality primarily take two forms (1) hand-crafted features, and (2) Deep Learning.

The early approaches were based on hand-crafted features, such as picking a specific texture and colour [19], SIFT descriptors [20], bag-of-colours [21] and then using SVM [22], KNN [23] as the classifier [24]. These approaches were limited by the choice of features, and limited accuracy [3]. Further, typically high computational costs inherently limit the size of the datasets used.

Chiang et al. use a dataset of 2,878 images to train a CNN classifier on 4 modalities [25], Abdominal CT, Brain CT, Lumbar Spine MRI, and Brain MRI, achieving an average validation accuracy of > 99.5%. Cheng et al. use a cascaded CNN to classify a bimodal dataset, comprised of MRI and PET images [26]. Using a dataset in the order of 102 images, they achieved a classification accuracy of 89.6%. Yu et al. use a DNN, and a dataset from the ImageCLEF database, comprising of 2,901 training and 2,582 test images to demonstrate a best classification accuracy of 70% [27]. Sevakula et al. use transfer learning to compare performance of seven DCNNs [28]. Using a curated dataset of 5,500 images from the Open-i Biomedical Image Search Engine, they achieve a best classification accuracy of 99.45% on the Inception-V3 network. Finally, Trenta et al. use a dataset comprised of 8,500 slices and a test set of 1,320 slices (split across 5 classes), and transfer learning techniques to achieve an overall accuracy of up to 100% on specific modalities, on their pre-trained VGGNet implementation [24].

EfficientNets use a set of heuristics, for constructing larger networks given an initial starting point, over a series of iterations [29]. A number of approaches using EfficientNets [30] for image classification were studied. In Nayak et al., the authors propose a CNN-based dense EfficientNet that uses min-max normalization to classify 3,260 T1-weighted contrast-enhanced brain magnetic resonance images into four categories (glioma, meningioma, pituitary, and no tumor). The model achieved a performance of 99.97% accuracy during training and 98.78% accuracy during testing [31]. Ali et al. use a dataset comprised of 10,015 images from the HAM10000 dataset to train a EfficientNet that achieves a Top-1 Accuracy of 87.91% [32]. On a smaller dataset size of about 3,500 images, Wang et al., use a Multi-Label Classification on Fundus Images to achieve an F1 score of 0.88 [33]. A relatively larger dataset of 33K images was used by Ha et al. In this implementation, diagnosis data and metadata were added to achieve an accuracy of 0.960AUC on cross validation [34]. This review of EfficientNet implementations seems to indicate that they are able to achieve fairly high classification accuracies. However, EfficientNet performance on larger dataset sizes seems relatively under-researched, and no data could be found on large (10^6^) dataset sizes.

Vision Transformers (ViT) use a transformer on sequences of image patches to classify the full image, achieving significant accuracy on a number of vision tasks [35]. A number of ViT implementations for classification were studied. Gheflati and Rivaz, use Vision Transformers for Classification of Breast Ultrasound Images, with weighted cross-entropy loss function to offset imbalances inherent in breast ultrasound datasets. They achieve an accuracy of 86% on a dataset size of 943 images [36]. Using a slightly larger dataset of 1,265 carcinoma clinical photographs, Flugge et al. [37], achieve classification accuracy of 0.986. In ViT implementations too, it is seen that research on large training datasets are sparse, with the largest dataset we were able to find being in the region of 10^4^ images [38], see Table 1.

**Table 1:** Dataset sizes vs Performance in previous literature.

Classifier	Study	Dataset Magnitude	Classifier Accuracy
CNN	Chiang et al. [25]	10^3	>99.5%
CNN	Cheng et al. [26]	10^2	>89.6%
DNN	Yu et al. [27]	10^3	70%
DCNN	Sevakula et al. [28]	10^3	99.45%
Transfer Learning	Trenta et al. [24]	10^3	100%
EfficientNets	Nayak et al. [31]	10^3	98.78%
EfficientNets	Ali et al. [32]	10^4	87.91%
EfficientNets	Wang et al. [33]	10^3	0.88 (F1 Score)
EfficientNets	Ha et al. [34]	10^4	0.96 (AUC)
ViT	Gheflati and Rivaz [36]	10^2	0.86 (AUC)
ViT	Flugge et al. [37]	10^3	0.986
ViT	Aldhadh et al. [38]	10^4	
ResNet3D	He et al. [39]	10^3	96% (F1 Score)

In a brief study of ResNet3D networks for classification, it is seen that datasets are the region of 10^3^ images. He et al. [39] use a dataset of 4,860 Optical Coherence Tomography (OCT) images to get a best model F1-score of 96%.

A number of approaches using deep learning classifiers are seen in literature. However, all approaches reviewed are seen to be utilising limited dataset volumes, with sizes in the (10^2^–10^4^) magnitude, typically hundreds to tens of thousands of images. Therefore, real-world classification performance of these algorithms, when operated on typical image-repository scales of millions of images seems unestablished.

To summarise, two findings emerge, (1) deep learning models present several advantages over handcrafted, feature driven models, and (2) it is seen that the largest of the datasets in the literature reviewed is in the order of 10^4^ images. Given that image repositories are now typically in the hyper-scale order, and growing rapidly, a suitably trained CNN capable of handling hyper-scale datasets is required.

## 2. Materials & methods

### 2.1. Data

**Fig 1:**
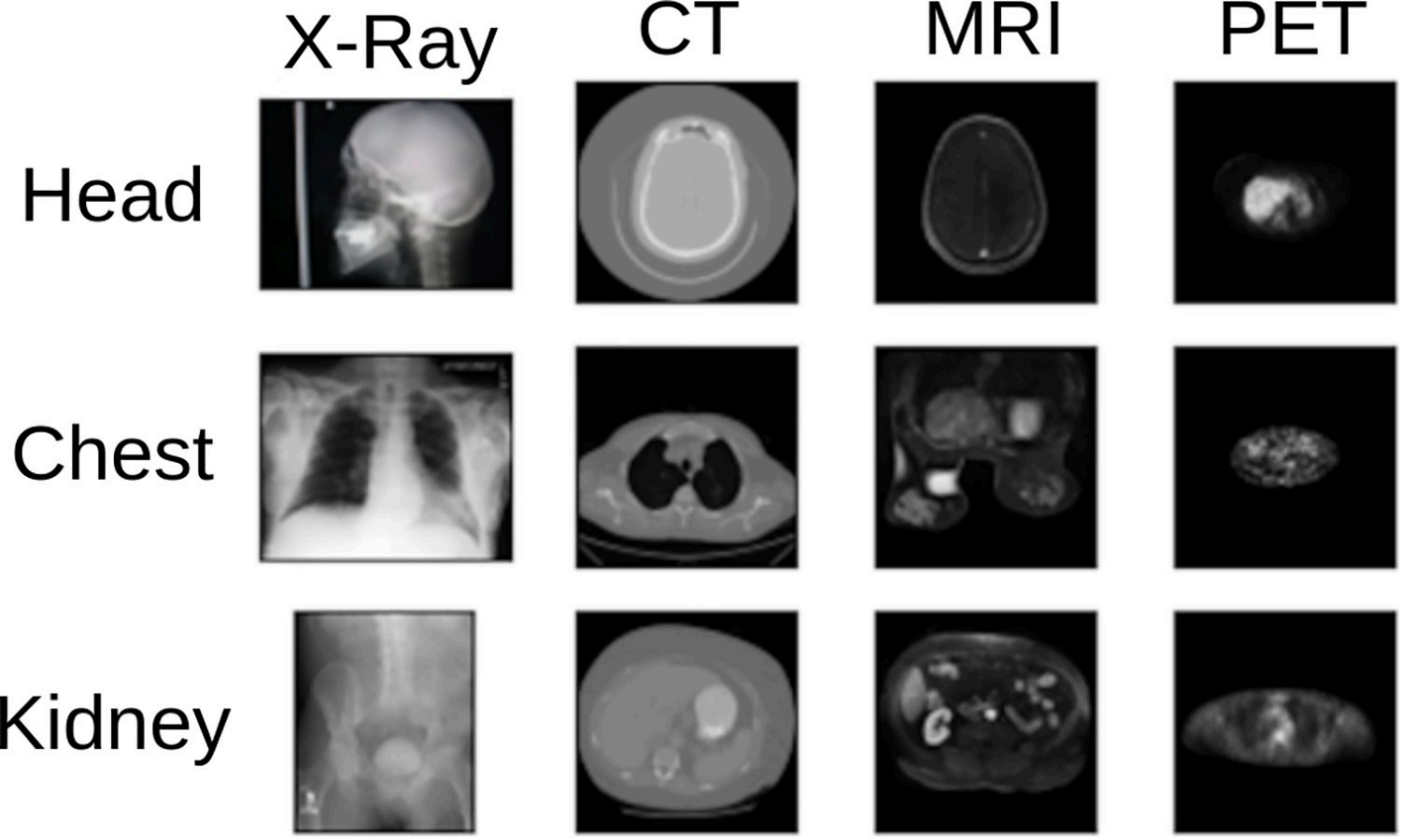
Visualisation of a spread of images from different locations in different modalities. Different modalities use different kinds of radiation, and these are absorbed to varying degrees by tissue in the human body. This leads to the same tissue looking different in each modality. Examples of modalities showing variation of the same tissue (left to right, top to bottom): [6–14,15].

In total, 102 datasets were downloaded and combined to form a hyper-scale image dataset of 4.5 million images. The full list of datasets with citations is provided in S1 Appendix. Four modalities were selected as targets for the classification task: CT, MRI, X-ray and PET (Fig 1). Other modalities (e.g. ultrasound) were excluded from this study because of a lack of appreciable volumes of data. The main source of this data was the Cancer Imaging Archive (TCIA) [16]. The Cancer Imaging Archive provides a REST API that allows for programmatic retrieval of images which allowed data to be downloaded and combined easily, and in a reproducible way. However, because the Cancer Imaging Archive’s main purpose is to host datasets relating to cancer research it was important to seek out some extra datasets to augment the data TCIA provides. The full list of datasets can be found in S1 Appendix.

This project was approved by the University of St Andrews University Teaching and Research Ethics Committee (UTREC), approval code CS15171.

### 2.2. Train-validate-test split

The downloaded data was split into three separate parts—train, validate and test. The train set was used to train the model, the validate set was used to evaluate the models between training runs, and the test set was used once to evaluate the final trained models. It was important to create the splits at the dataset level to prevent data-leakage. That is, all the images from a dataset were placed in the same split. Scans of the same patient in the same modality are likely to be similar, so if there is an image of the same patient in the train and test set then the test set does not contain completely unseen data. Putting each dataset into one of train, validate or test prevents this data leakage. Splitting the datasets like this also helps achieve the goal of demonstrating generalisation across datasets, because no dataset in the train set is represented in the test set.

**Fig 2:**
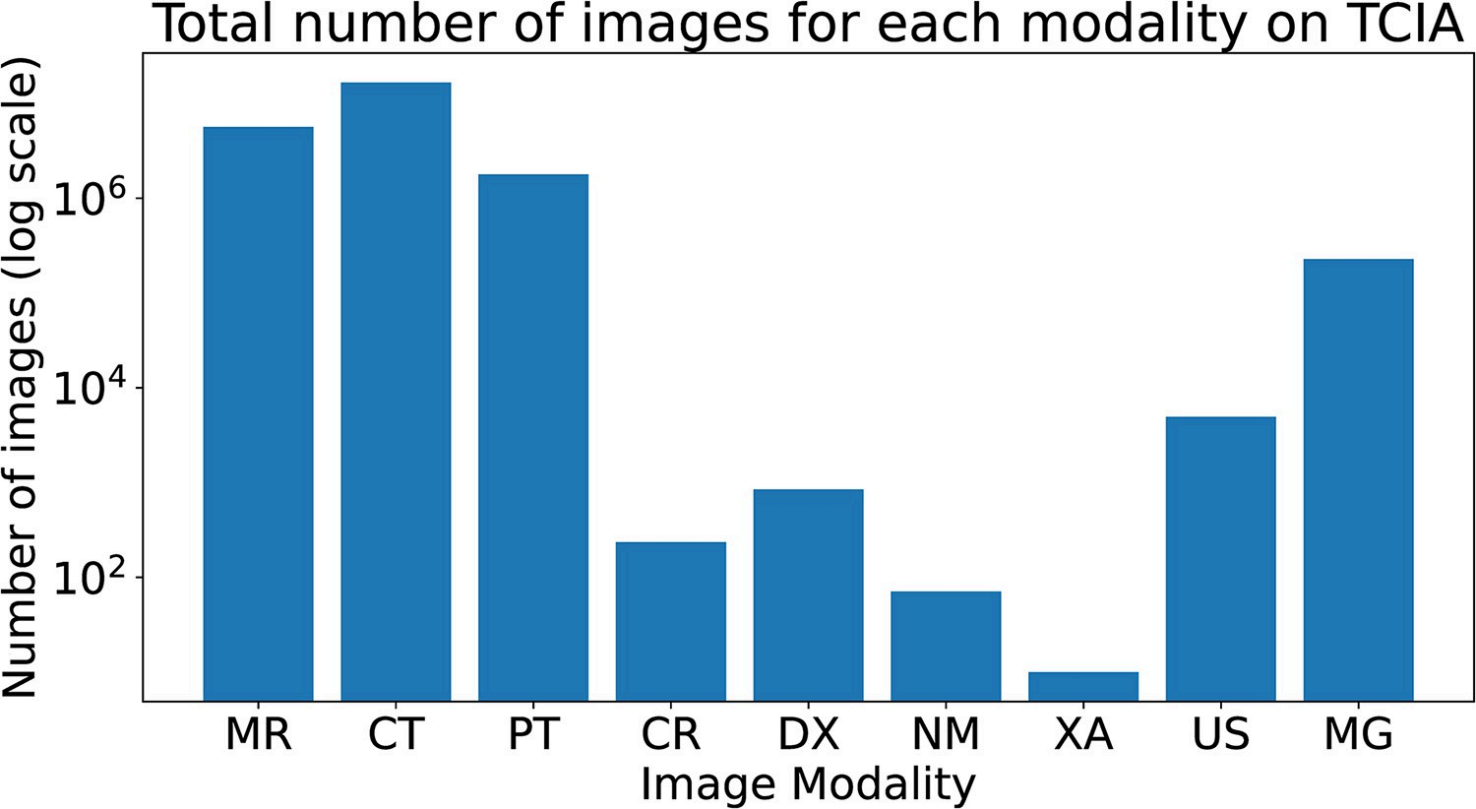
Distribution of Image Modalities.

**Fig 3:**
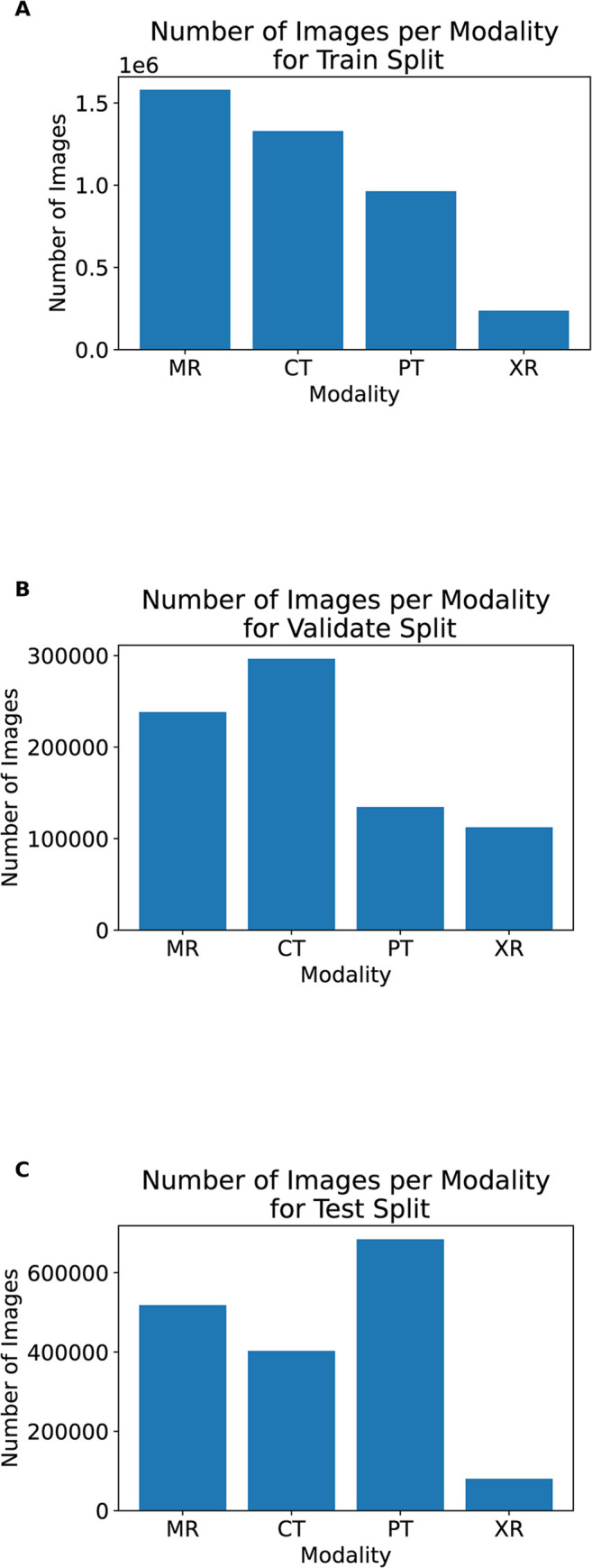
Figures showing the number of images for each modality in the created splits: A) train, B) validate and C) test. Note that each graph has a different scale, the purpose is to show the ratios of each class are similar. There are 73 datasets in the train set, 13 in the validate set and 16 in the test set.

The train-validate-test split was created manually to ensure as even a spread as possible of images for each modality and location in each split. The manual split ensured that there are at least two locations for each modality in each of the train, validate and test split. The main difficulty for this was X-rays, because in the TCIA datasets most X-rays are mammograms (Fig 2). This meant the non-TCIA datasets had to be carefully split. Again, the table in S1 Appendix shows the split each dataset was placed in. Fig 3 shows the number of images in the train, validate and test set. TCIA hosts many CT and MR datasets and some of these datasets are very large. For example, the CT Colonography dataset [40] has more than 900,000 CT images, which is more than the total number of X-ray images across all datasets used in this study. To ensure the other modalities were not completely dwarfed by these datasets, a maximum of 50,000 CT images and 100,000 MR images was taken from each individual dataset. The images were selected in the order given by TCIA. This selection method was not applied to the images from sources other than TCIA. After imbalance correction, the total number of images in the dataset were 6,433,838 (6.4 million images), with a spilt of 4,104,184 in training, 936,347 in test, and 1,393,307 in validate datasets.

### 2.3. Preprocessing

In order for 2D and 3D scans to be used in the same study, the 3D scans (CT, MR and PT) were treated as a collection of 2D images. These images are sometimes referred to as slices. The images were resized to 224×224 and rescaled between 0 and 1. Each image was rescaled using min-max normalisation with the maximum and minimum values being the highest and lowest pixel values present within the image.

### 2.4. Network architecture and training

The models trained on this dataset were a ResNet-18 [41], ResNet-50 and a VGG16 [42]. The code used was adapted from PyTorch’s hosted versions of these models [43]. Changes were made to the channel depth of the input layer, from three channels to one channel (grayscale). These three models were chosen because they have all been shown to perform well when trained with large quantities of data on the ImageNet dataset [41,42]. The code created as part of this research is open-source and hosted online at GitHub [44].

All models were trained for 10 epochs with a batch size of 128. The training set contained 2,954,097 (2.9×10^6^) samples and the validate set contained 704,685 samples. The models were optimised using stochastic gradient descent, with a learning rate of 0.1 that was divided by 10 every time the loss plateaued, a momentum of 0.9 and an L2 weight decay penalty of 0.005. The models were trained on a machine with an Intel(R) Xeon(R) CPU E5-1650 v4 @3.60GHz with 6 physical cores (12 threads), 250GB of RAM and two Nvidia GeForce GTX 1080Tis.

### 2.5. Transfer learning

As this model was trained on 4 million images, the model’s ability to perform feature extraction on unseen medical images of the human body was tested, i.e. validate performance of the saved weights for transfer learning on a different medical imaging task.

The MURA (MUsculoskeletal RAdiographs) dataset [45] was chosen as it was the only labelled X-Ray dataset in the test set. To test this hypothesis, the MURA dataset [45] was used for the transfer learning task. The MURA dataset contains 40,561 X-Ray images labelled as “normal” or “abnormal” in the opinion of multiple board-certified radiologists.

With the aim of testing our model as a foundation model, we took the saved weights of the ResNet50 from our original task and added a new binary classification head. This model was compared to a model with the same architecture but with randomly initialised weights.

The pretrained model was first trained with only the dense classification layers being trainable for 40 epochs with a learning rate of 1×10^−1^ to 1×10^−4^, then the remaining layers were set to trainable and trained at a learning rate of 1×10^−4^ decaying to 1×10^−8^ for 100 epochs. The model trained from scratch was trained for 100 epochs with a learning rate starting at 1×10^−2^ and decaying to 1×10^−6^.

## 3. Results & discussion

### 3.1. Training and validation accuracy

**Fig 4:**
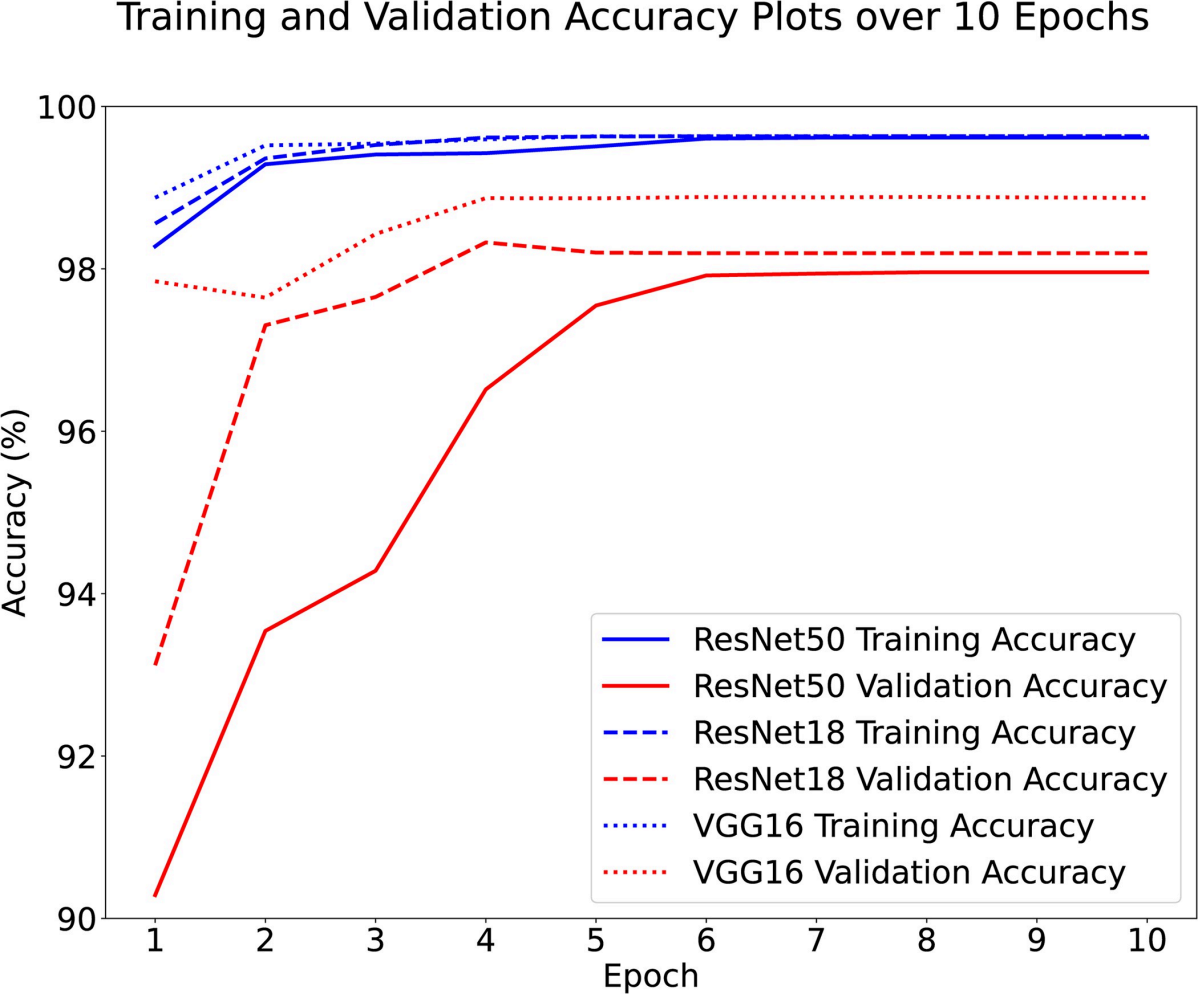
Training and validation accuracy each of the three networks, found at the end of each epoch. The small gap between the training and validation accuracies suggests that the models are not overfitting. Note the scale starts at 90%.

**Fig 5:**
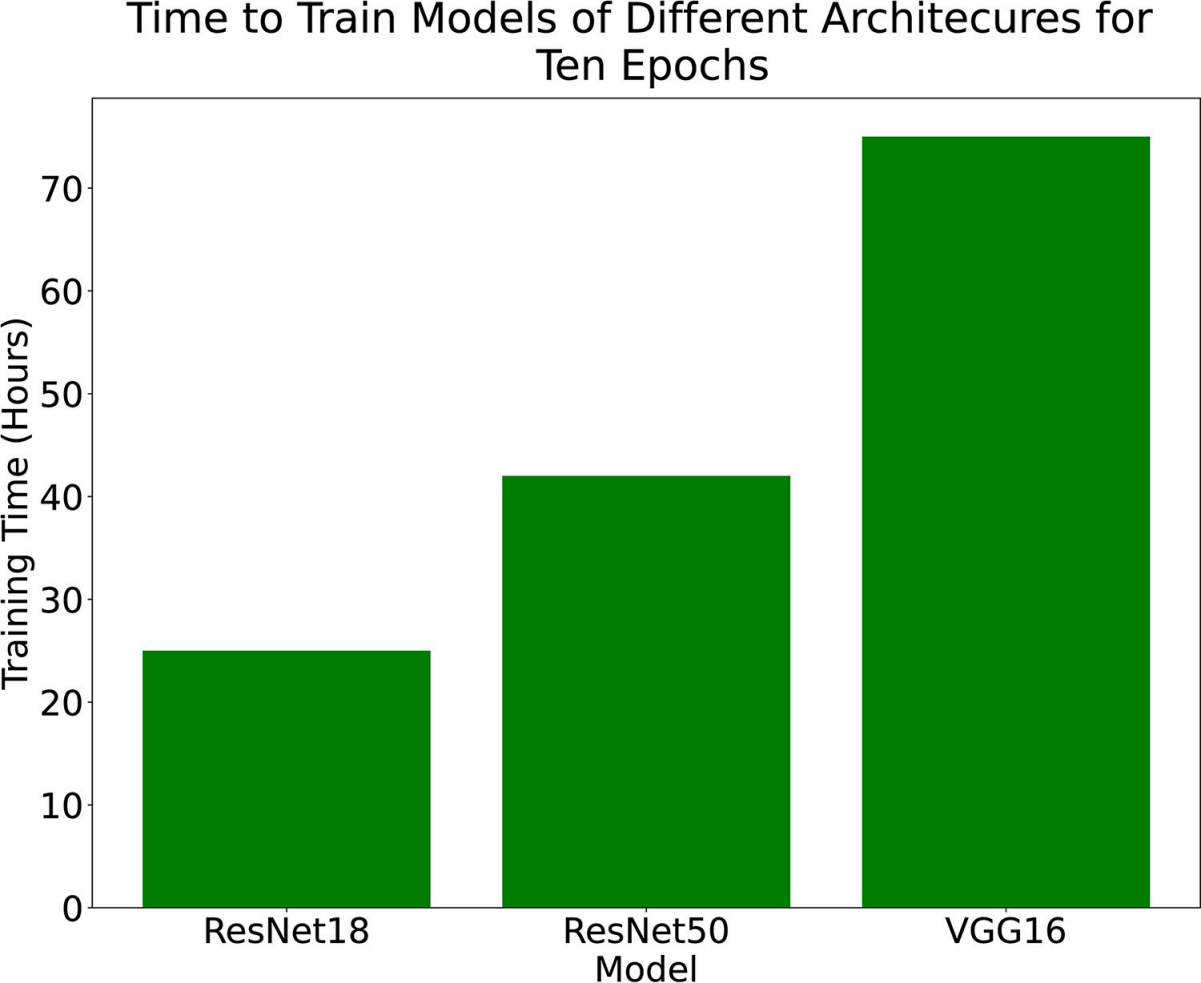
Time in hours to train the models for 10 epochs. The training and validation accuracy both level-off around epochs 5–6 which shows that the models are able to fit the data.

Fig 4 shows the training and validation accuracy curves for the ResNet50, ResNet18 and VGG16 models. The small gap between the training and validation accuracies suggests that the models are not overfitting. Fig 5 shows the time it took to train the models over the 10 epochs.

### 3.2. Test set accuracy

**Fig 6:**
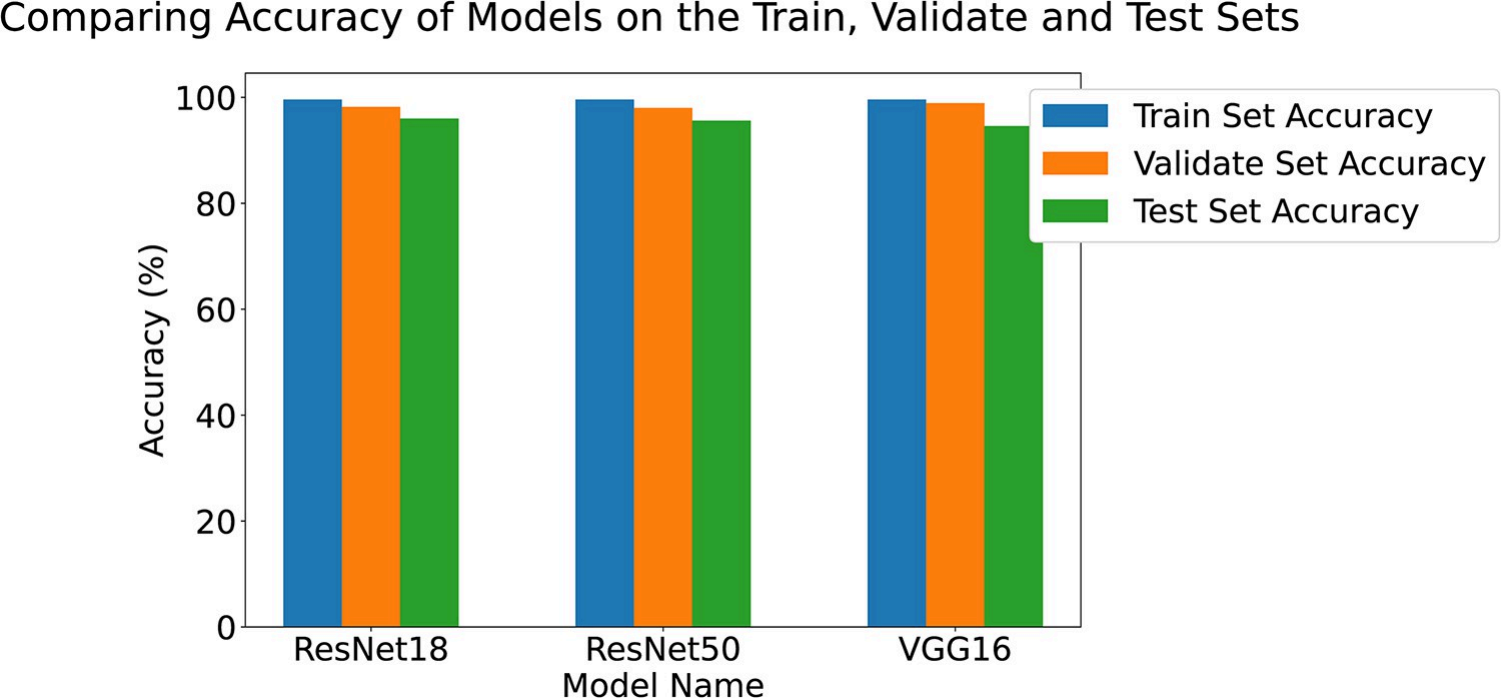
Accuracy of 3 models on the test set.

**Fig 7:**
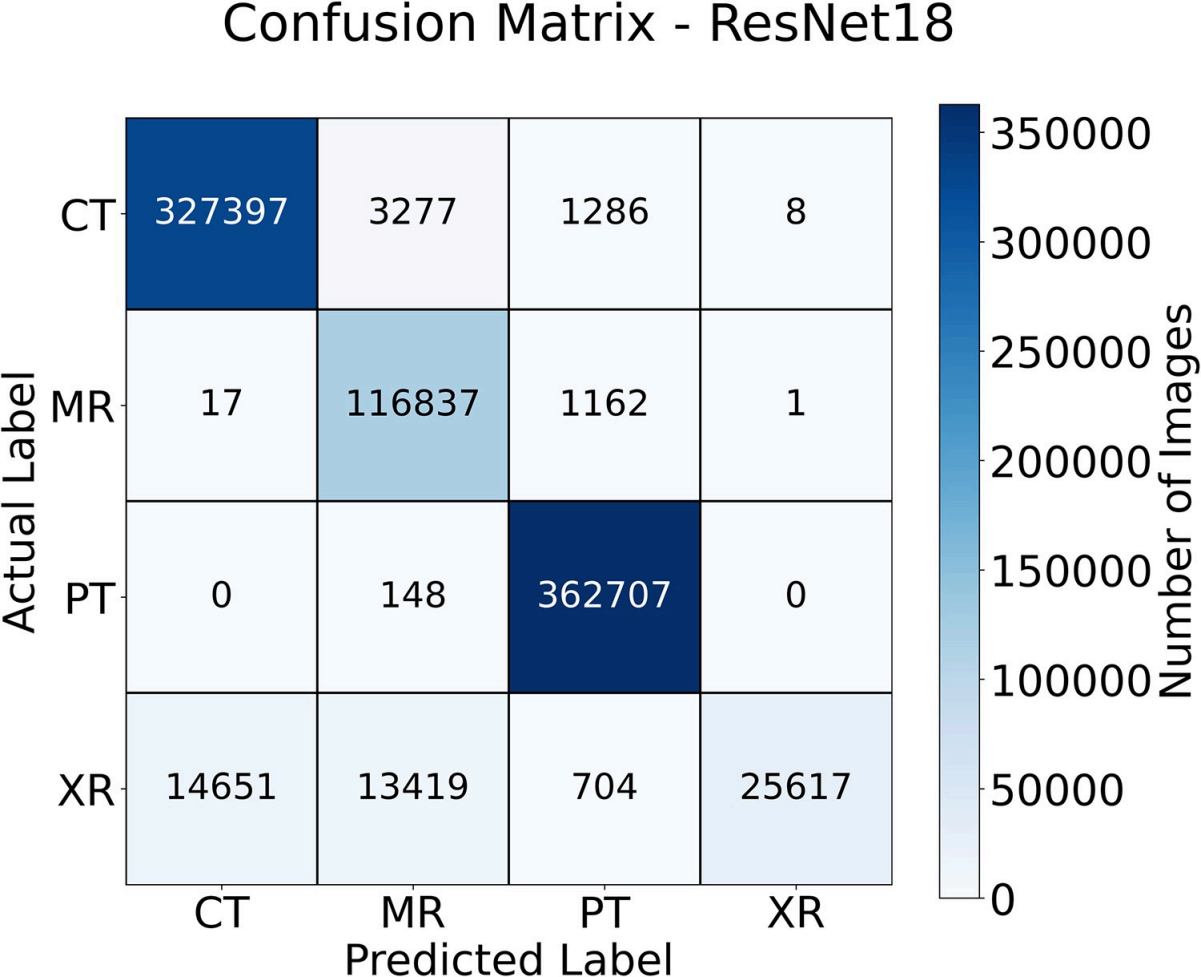
The confusion matrix for the ResNet18 on the test set. The model gains very high accuracy on the CT, MRI and PET. The ResNet18 results were chosen for this plot as this model achieved the highest accuracy and highest balanced accuracy.

Fig 6 shows the accuracy of the three models. These results are in the high 90%’s across the train, validate and test sets which shows that the models have all learned the problem well. Table 2 shows the accuracy and balanced accuracy of each of the models on the test set. Tables 3, 4, and 5 show the per-class precision, recall, F1 Score and AUROC for the ResNet50, ResNet18 and VGG16 respectively. Fig 7 shows the confusion matrix for the ResNet18 model. The confusion matrix shows that the model performs very well on CT, MR and PET. Accuracy for X-rays can be improved by adding additional X-ray images across a larger spread of locations.

**Table 2:** Table containing the accuracy and balanced accuracy of various models on the test set. Each model was trained for 10 epochs.

Model	Accuracy	Balanced Accuracy
ResNet18	96.00%	86.17%
ResNet50	95.60%	85.65%
VGG16	94.58%	81.08%

**Table 3:** ResNet 50 Metrics, Average AUC = 0.9971405583309333.

Class	Precision	Recall	F1	One vs Rest AUROC
CT	0.95197	0.97861	0.96511	0.99697802
MR	0.86901	0.98843	0.92488	0.99818678
PT	0.9885	0.99899	0.99372	0.9999379
XR	0.99944	0.45998	0.63001	0.99345952

**Table 4:** ResNet 18 Metrics, Average AUC = 0.9976741643022355.

Class	Precision	Recall	F1	One vs Rest AUROC
CT	0.95712	0.98623	0.97146	0.99791051
MR	0.874	0.99	0.92839	0.99840761
PT	0.99138	0.99959	0.99547	0.99997337
XR	0.99965	0.47098	0.64029	0.99440516

**Table 5:** VGG16 Metrics, Average AUC = 0.9987707721217087.

Class	Precision	Recall	F1	One vs Rest AUROC
CT	0.91695	0.98165	0.94819	0.99782978
MR	0.88167	0.99672	0.93567	0.99908888
PT	0.99526	0.99814	0.9967	0.99992527
XR	0.99924	0.26675	0.42109	0.99823916

### 3.3. Dataset level results

Table 6 shows the accuracy of the model on each dataset in the test set for the ResNet18 model, chosen because this model demonstrated superior classification performance over others tested in this study. It is interesting to note that in both tables the X-ray performance is in the 80–90% range for the Cancer Imaging Archive X-ray datasets, then drops for the MURA and Osteoarthritis Initiative datasets. This is likely because these datasets are bone X-rays, and most of the datasets only contain chest X-rays. Therefore, a better spread of X-ray datasets is needed for the performance of these models to be improved.

**Table 6:** Table containing the accuracy of the ResNet18 model on every dataset in the test set. Some datasets appear more than once in this table because they contain multiple image modalities.

Dataset (Location)	Modality	Accuracy (%)
CPTAC-LUAD (Chest)	CT	99
Pelvic-Reference-Data(Pelvis)	CT	81
C4KC-KiTS (Kidney)	CT	100
Anti-PD-1 Lung (Chest)	CT	97
CPTAC-PDA (Pancreas)	CT	100
NaF PROSTATE (Prostate)	CT	100
TCGA-READ (Kidney)	CT	100
QIN-HEADNECK (Head)	CT	100
CPTAC-LSCC (Chest)	CT	100
CPTAC-CCRCC (Kidney)	CT	100
CPTAC-LUAD (Chest)	MR	100
ISPY1 (Breast)	MR	99
Brain-Tumor-Progression (Head)	MR	92
REMBRANDT (Head)	MR	100
BraTS20 (Head)	MR	97
CPTAC-PDA (Pancreas)	MR	99
TCGA-READ (Kidney)	MR	98
CPTAC-CCRCC (Kidney)	MR	99
CPTAC-LUAD (Chest)	PT	100
Anti-PD-1 Lung (Chest)	PT	100
QIN-HEADNECK (Head)	PT	100
CPTAC-PDA (Pancreas)	PT	100
NaF PROSTATE (Prostate)	PT	100
CPTAC-LSCC (Chest)	PT	100
CPTAC-LUAD (Chest)	XR	100
CPTAC-PDA (Pancreas)	XR	96
CPTAC-LSCC (Chest)	XR	92
CPTAC-CCRCC (Kidney)	XR	100
MURA (Bone)	XR	28
Osteo-Arthritis Initiative (Bone)	XR	62

### 3.4. Transfer learning results

The developed model’s ability to perform feature extraction on unseen medical images of the human body was tested, i.e. validate performance of the saved weights for transfer learning on a different medical imaging task.

To test this hypothesis, the MURA dataset (MUsculoskeletal RAdiographs) [45] was used for the transfer learning task. The MURA dataset contains 40,561 X-Ray images labelled as “normal” or “abnormal” in the opinion of multiple board-certified Stanford radiologists.

The results shown in Table 7 indicate that the model trained from scratch on disease detection tasks demonstrated 77.7% accuracy in detecting abnormal X-ray images, while the model with transfer learning weights showed marginal improvements, with 78% accuracy, in detecting abnormal X-ray images. Tables 8 and 9, indicate the precision, recall, F1 Score and AUROC for the ResNet50 model trained from scratch and pretrained model respectively. These results are hypothesised to be due to the following factors:

1. limited X-ray images in the modality distribution, i.e. the pre-trained model is fitted to modalities that are abundant in the training dataset, such as MRI, CT, and PT.
2. Transferred weights and parameters might need further tweaking [46] to generalise better to work with sparse modalities, such as X-Rays, as tested in this case.

**Table 7:** Transfer Learning Results on X-Ray Image Classification.

Model	Accuracy
Our Pretrained ResNet50	78.04
ResNet50 Trained from Scratch	77.73

**Table 8:** ResNet50 Trained from Scratch, AUROC = 0.8470.

Class	Precision	Recall	F1 Score
Normal	0.7328	0.9016	0.8084
Abnormal	0.8568	0.6418	0.7339

**Table 9:** Pretrained ResNet50, AUROC = 0.8514.

Class	Precision	Recall	F1 Score
Normal	0.7462	0.8770	0.8063
Abnormal	0.8344	0.6751	0.7463

## 4. Conclusion

In this work, we proposed a hyper-scale classifier, capable of classifying diagnostic imaging data in the scale of millions of medical images, with significant classification accuracy. We used a dataset comprised of 4.5 million images to train a ResNet-50, ResNet-18, and VGG16 CNN. The trained classifiers were then tested for their classification accuracy on 4 modalities (Computed Tomography (CT), Magnetic Resonance Imaging (MRI), Positron Emission Tomography (PET), and X-ray). The best performing model demonstrated a classification accuracy of 96%. Our results show that CNN-based hyper-scale classifiers are capable of accurately classifying volumes of image data encountered in real-word applications, such as those contained in image repositories or diagnostic imaging data collected by national healthcare institutions.

The classification accuracy of this proposed hyper-scale classifier is comparable to or exceeds the accuracy of more complex classifier implementations that use EfficientNet or Vision Transformers (ViT).

Future work on this topic will include extending the scope of the hyper-scale modality classifier to work on 3D scan modalities, such as CT, MR, and PET.

## Supporting information

S1 Appendix List of all datasets used, resource location, and how used, i.e. train, validate, or test datasets. (DOCX)
