# Supplementary material for: Classification of hyper-scale multimodal imaging datasets
Source: PLOS Digit Health. 2023 Dec 13;2(12):e0000191. doi: 10.1371/journal.pdig.0000191 (PMC10718410; doi:10.1371/journal.pdig.0000191)
Supplement: S1 Appendix — (DOCX) [file pdig.0000191.s001.docx]

| Name | Dataset Webpage | Split | Citations |
| --- | --- | --- | --- |
| ACRIN-DSC-MR-Brain | https://doi.org/10.7937/tcia.2019.zr1pjf4i | Train | 2 |
| Head-Neck-Radiomics-HN1 | https://doi.org/10.7937/tcia.2019.8kap372n | Train | 13 |
| Lung-PET-CT-Dx | https://doi.org/10.7937/TCIA.2020.NNC2-0461 | Train | 5 |
| AAPM RT-MAC Grand Ch. 2019 | https://doi.org/10.7937/tcia.2019.bcfjqfqb | Train | 14 |
| COVID-19-AR | https://doi.org/10.7937/tcia.2020.py71-5978 | Train | 15 |
| CPTAC-CM | https://doi.org/10.7937/K9/TCIA.2018.ODU24GZE | Train | 8 |
| CPTAC-HNSCC | https://doi.org/10.7937/K9/TCIA.2018.UW45NH81 | Train | 16 |
| PDMR-997537-175-T | https://doi.org/10.7937/TCIA.2020.BRY9-4N29 | Train | 17 |
| PDMR-292921-168-R | https://doi.org/10.7937/TCIA.2020.PCAK-8Z10 | Train | 17 |
| PDMR-425362-245-T | https://doi.org/10.7937/TCIA.2020.7YRS-7J97 | Train | 17 |
| HNSCC | https://doi.org/10.7937/k9/tcia.2020.a8sh-7363 | Train | 18.19 |
| DRO Toolkit | https://doi.org/10.7937/t062-8262 | Train | 20 |
| QIN GBM Treatment Response | https://doi.org/10.7937/K9/TCIA.2016.nQF4gpn2 | Train | 21 |
| CPTAC-GBM | https://doi.org/10.7937/K9/TCIA.2018.3RJE41Q1 | Train | 22 |
| CPTAC-SAR | https://doi.org/10.7937/TCIA.2019.9bt23r95 | Train | 23 |
| CPTAC-UCEC | https://doi.org/10.7937/K9/TCIA.2018.3R3JUISW | Train | 24 |
| OPC-Radiomics | https://doi.org/10.7937/tcia.2019.8dho2gls | Train | 25 |
| Acrin-FLT-Breast (ACRIN 6688) | https://doi.org/10.7937/K9/TCIA.2017.ol20zmxg | Train | 3 |
| QIN-Breast | https://doi.org/doi:10.7937/K9/TCIA.2016.21JUebH0 | Train | 26 |
| Lung Fused-CT-Pathology | https://doi.org/10.7937/K9/TCIA.2018.SMT36LPN | Train | 27 |
| NSCLC-Radiomics | https://doi.org/10.7937/K9/TCIA.2015.PF0M9REI | Train | 13 |
| NSCLC-Radiomics-Interobserver1 | https://doi.org/10.7937/tcia.2019.cwvlpd26 | Train | 13,28 |
| PDMR-BL0293-F563 | https://doi.org/10.7937/tcia.2019.b6u7wmqw | Train | 17 |
| QIN-BRAIN-DSC-MRI | https://doi.org/doi:10.7937/K9/TCIA.2016.5DI84Js8 | Train | 29 |
| CC-Radiomics-Phantom | https://doi.org/10.7937/K9/TCIA.2017.zuzrml5b | Train | 30 |
| CC-Radiomics-Phantom-2 | https://doi.org/10.7937/TCIA.2019.4l24tz5g | Train | 30 |
| CC-Radiomics-Phantom-3 | https://doi.org/10.7937/tcia.2019.j71i4fah | Train | 30 |
| LCTSC | https://doi.org/10.7937/K9/TCIA.2017.3r3fvz08 | Train | 31 |
| Anti-PD-1 MELANOMA | https://doi.org/10.7937/tcia.2019.1ae0qtcu | Train | 32 |
| TCGA-UCEC | https://doi.org/10.7937/K9/TCIA.2016.GKJ0ZWAC | Train | 33 |
| TCGA-HNSC | https://doi.org/10.7937/K9/TCIA.2016.LXKQ47MS | Train | 34 |
| HNSCC-3DCT-RT | https://doi.org/10.7937/K9/TCIA.2018.13upr2xf | Train | 35 |
| MRI-DIR | https://doi.org/10.7937/K9/TCIA.2018.3f08iejt | Train | 36 |
| Head-Neck-PET-CT | https://doi.org/10.7937/K9/TCIA.2017.8oje5q00 | Train | 37 |
| LGG-1p19qDeletion | https://doi.org/10.7937/K9/TCIA.2017.dwehtz9v | Train | 38 |
| CBIS-DDSM | https://doi.org/10.7937/K9/TCIA.2016.7O02S9CY | Train | 39 |
| Phantom FDA | https://doi.org/10.7937/K9/TCIA.2015.ORBJKMUX | Train | 40 |
| QIN LUNG CT | https://doi.org/10.7937/K9/TCIA.2015.NPGZYZBZ | Train | 41 |
| Mouse-Astrocytoma | https://doi.org/10.7937/K9TCIA.2017.SGW7CAQW | Train | 42 |
| TCGA-LUSC | https://doi.org/10.7937/K9/TCIA.2016.TYGKKFMQ | Train | 43 |
| TCGA-LUAD | https://doi.org/10.7937/K9/TCIA.2016.JGNIHEP5 | Train | 43 |
| TCGA-KIRP | https://doi.org/10.7937/K9/TCIA.2016.ACWOGBEF | Train | 10 |
| TCGA-LIHC | https://doi.org/10.7937/K9/TCIA.2016.IMMQW8UQ | Train | 44 |
| IvyGAP | https://doi.org/10.7937/K9/TCIA.2016.XLwaN6nL | Train | 45 |
| Prostate Fused-MRI-Pathology | https://doi.org/10.7937/K9/TCIA.2016.TLPMR1AM | Train | 45-49 |
| TCGA-PRAD | https://doi.org/10.7937/K9/TCIA.2016.YXOGLM4Y | Train | 50 |
| Breast-MRI-NACT-Pilot | https://doi.org/10.7937/K9/TCIA.2016.QHSYHJKY | Train | 51 |
| RIDER Neuro MRI | https://doi.org/10.7937/K9/TCIA.2015.VOSN3HN1 | Train | 52 |
| Soft-tissue-Sarcoma | https://doi.org/10.7937/K9/TCIA.2015.7GO2GSKS | Train | 53 |
| Mouse-Mammary | https://doi.org/10.7937/K9/TCIA.2015.9P42KSE6 | Train | 54 |
| TCGA-THCA | https://doi.org/10.7937/K9/TCIA.2016.9ZFRVF1B | Train | 55 |
| TCGA-SARC | https://doi.org/10.7937/K9/TCIA.2016.CX6YLSUX | Train | 56 |
| LungCT-Diagnosis | https://doi.org/10.7937/K9/TCIA.2015.A6V7JIWX | Train | 57 |
| TCGA-CESC | https://doi.org/10.7937/K9/TCIA.2016.SQ4M8YP4 | Train | 58 |
| TCGA-OV | https://doi.org/10.7937/K9/TCIA.2016.NDO1MDFQ | Train | 59 |
| TCGA-COAD | https://doi.org/10.7937/K9/TCIA.2016.HJJHBOXZ | Train | 55 |
| TCGA-KIRC | https://doi.org/10.7937/K9/TCIA.2016.V6PBVTDR | Train | 9 |
| TCGA-LGG | https://doi.org/10.7937/K9/TCIA.2016.L4LTD3TK | Train | 60 |
| QIN PET Phantom | https://doi.org/10.7937/K9/TCIA.2015.ZPUKHCKB | Train | 61 |
| QIN Breast DCE-MRI | https://doi.org/10.7937/K9/TCIA.2014.A2N1IXOX | Train | 62 |
| NSCLC-Radiomics-Genomics | https://doi.org/10.7937/K9/TCIA.2015.L4FRET6Z | Train | 13 |
| Lung Phantom | https://doi.org/10.7937/K9/TCIA.2015.08A1IXOO | Train | 63 |
| TCGA-KICH | https://doi.org/10.7937/K9/TCIA.2016.YU3RBCZN | Train | 64 |
| TCGA-GBM | https://doi.org/10.7937/K9/TCIA.2016.RNYFUYE9 | Train | 65 |
| SPIE-AAPM Lung CT Challenge | https://doi.org/10.7937/K9/TCIA.2015.UZLSU3FL | Train | 66 |
| Prostate-3T | https://doi.org/10.7937/K9/TCIA.2015.QJTV5IL5 | Train | 67 |
| Prostate-Diagnosis | https://doi.org/10.7937/K9/TCIA.2015.FOQEUJVT | Train | 68 |
| RIDER Phantom PET-CT | https://doi.org/10.7937/K9/TCIA.2015.8WG2KN4W | Train | 69 |
| RIDER Lung CT | https://doi.org/10.7937/K9/TCIA.2015.U1X8A5NR | Train | 70 |
| RIDER Phantom MRI | https://doi.org/10.7937/K9/TCIA.2015.MI4QDDHU | Train | 71 |
| RIDER Breast MRI | https://doi.org/10.7937/K9/TCIA.2015.H1SXNUXL | Train | 72 |
| CT Colonography (ACRIN 6664) | https://doi.org/10.7937/K9/TCIA.2015.NWTESAY1 | Train | 11 |
| Chexpert | https://stanfordmlgroup.github.io/competitions/chexpert/ | Train | 4 |
| RSNA Bone Age | https://www.kaggle.com/kmader/rsna-bone-age | Train | None |
| TCGA-BRCA | https://doi.org/10.7937/K9/TCIA.2016.AB2NAZRP | Validate | 6 |
| Acrin-FMISO-Brain (ACRIN 6684) | https://doi.org/10.7937/K9/TCIA.2018.vohlekok | Validate | 1 |
| TCGA-BLCA | https://doi.org/10.7937/K9/TCIA.2016.8LNG8XDR | Validate | 55 |
| Pancreas-CT | https://doi.org/10.7937/K9/TCIA.2016.tNB1kqBU | Validate | 73 |
| CT Lymph Nodes | https://doi.org/10.7937/K9/TCIA.2015.AQIIDCNM | Validate | 74 |
| TCGA-ESCA | https://doi.org/10.7937/K9/TCIA.2016.VPTNRGFY | Validate | 75 |
| TCGA-STAD | https://doi.org/10.7937/K9/TCIA.2016.GDHL9KIM | Validate | 76 |
| LIDC-IDRI | https://doi.org/10.7937/K9/TCIA.2015.LO9QL9SX | Validate | 77 |
| QIBA CT-1C | https://doi.org/10.7937/K9/TCIA.2016.YxgR4blU | Validate | 78 |
| RIDER Lung PET-CT | https://doi.org/10.7937/K9/TCIA.2015.OFIP7TVM | Validate | 7 |
| Prostate-MRI | https://doi.org/10.7937/K9/TCIA.2016.6046GUDv | Validate | 79 |
| NIH 100000 Chest X-ray | https://nihcc.app.box.com/v/ChestXray-NIHCC | Validate | 80 |
| MRNet: Knee MRIs | https://stanfordmlgroup.github.io/competitions/mrnet/ | Validate | 81 |
| CPTAC-CCRCC | https://doi.org/10.7937/K9/TCIA.2018.OBLAMN27 | Test | 82 |
| C4KC-KiTS | https://doi.org/10.7937/TCIA.2019.IX49E8NX | Test | 83 |
| CPTAC-LSCC | https://doi.org/10.7937/K9/TCIA.2018.6EMUB5L2 | Test | 84 |
| LDCT-and-Projection-data | https://doi.org/10.7937/9npb-2637 | Test | 85 |
| CPTAC-LUAD | https://doi.org/10.7937/K9/TCIA.2018.PAT12TBS | Test | 86 |
| CPTAC-PDA | https://doi.org/10.7937/K9/TCIA.2018.SC20FO18 | Test | 87 |
| Pelvic-Reference-Data | https://doi.org/10.7937/TCIA.2019.woskq5oo | Test | 88 |
| Anti-PD-1 Lung | https://doi.org/10.7937/tcia.2019.zjjwb9ip | Test | 89 |
| ISPY1 (ACRIN 6657) | https://doi.org/10.7937/K9/TCIA.2016.HdHpgJLK | Test | 90 |
| QIN-HeadNeck | https://doi.org/10.7937/K9/TCIA.2015.K0F5CGLI | Test | 91 |
| TCGA-READ | https://doi.org/10.7937/K9/TCIA.2016.F7PPNPNU | Test | 92 |
| NaF Prostate | https://doi.org/10.7937/K9/TCIA.2015.ISOQTHKO | Test | 93 |
| REMBRANDT | https://doi.org/10.7937/K9/TCIA.2015.588OZUZB | Test | 94 |
| MURA | https://stanfordmlgroup.github.io/competitions/mura/ | Test | 12 |
| NDA Osteoarthritis Initiative | https://nda.nih.gov/oai/ | Test | None |
| BraTS20 | http://braintumorsegmentation.org/ | Test | None |

References:

1. Gerstner ER, Zhang Z, Fink JR, Muzi M, Hanna L, Greco E, et al. ACRIN 6684: assessment of tumor hypoxia in newly diagnosed glioblastoma using 18F-FMISO PET and MRI. Clinical Cancer Research. 2016;22(20):5079–5086.
2. Boxerman JL, Zhang Z, Safriel Y, Larvie M, Snyder BS, Jain R, et al. Early post-bevacizumab progression on contrast-enhanced MRI as a prognostic marker for overall survival in recurrent glioblastoma: results from the ACRIN 6677/RTOG 0625 Central Reader Study. Neuro-Oncology. 2013 07;15(7):945–954.
3. Kostakoglu L, Duan F, Idowu MO, Jolles PR, Bear HD, Muzi M, et al. A phase II study of 3’-deoxy-3’-18Ffluorothymidine PET in the assessment of early response of breast cancer to neoadjuvant chemotherapy: results from ACRIN 6688. Journal of Nuclear Medicine. 2015;56(11):1681–1689.
4. Irvin J, Rajpurkar P, Ko M, Yu Y, Ciurea-Ilcus S, Chute C, et al. CheXpert: A Large Chest Radiograph Dataset with Uncertainty Labels and Expert Comparison. CoRR. 2019;abs/1901.07031.
5. Li P, Wang S, Li T, Lu J, HuangFu Y, Wang D. A Large-Scale CT and PET/CT Dataset for Lung Cancer Diagnosis [Data set]. The Cancer Imaging Archive. 2020.
6. Lingle W, Erickson B, Zuley M, Jarosz R, Bonaccio E, Filippini J, et al. Radiology data from the cancer genome atlas breast invasive carcinoma [tcga-brca] collection. The Cancer Imaging Archive. 2016.
7. Muzi P, Wanner M, Kinahan P. Data From RIDER Lung PET-CT. The Cancer Imaging Archive. 2015.
8. Consortium NCICPTA. Radiology Data from the Clinical Proteomic Tumor Analysis Consortium Cutaneous Melanoma [CPTAC-CM] collection. The Cancer Imaging Archive. 2018.
9. Akin O, Elnajjar P, Heller M, Jarosz R, Erickson B, Kirk S, et al. Radiology data from the cancer genome atlas kidney renal clear cell carcinoma [TCGA-KIRC] collection. The Cancer Imaging Archive. 2016.
10. Linehan M, Gautam R, Kirk S, Lee Y, Roche C, Bonaccio E, et al. Radiology data from the cancer genome atlas cervical kidney renal papillary cell carcinoma [KIRP] collection. Cancer Imaging Arch. 2016.
11. Johnson CD, Chen MH, Toledano AY, Heiken JP, Dachman A, Kuo MD, et al. Accuracy of CT colonography for detection of large adenomas and cancers. New England Journal of Medicine. 2008; 359(12):1207–1217.
12. Rajpurkar P, Irvin J, Bagul A, Ding D, Duan T, Mehta H, et al. Mura: Large dataset for abnormality detection in musculoskeletal radiographs. arXiv preprint arXiv:171206957. 2017.
13. Aerts HJ, Velazquez ER, Leijenaar RT, Parmar C, Grossmann P, Carvalho S, et al. Decoding tumour phenotype by noninvasive imaging using a quantitative radiomics approach. Nature communications. 2014;5(1):1–9.
14. Cardenas CE, Mohamed AS, Yang J, Gooding M, Veeraraghavan H, Kalpathy-Cramer J, et al. Head and neck cancer patient images for determining auto-segmentation accuracy in T2-weighted magnetic resonance imaging through expert manual segmentations.Medical Physics. 2020;47(5):2317–2322.
15. Desai S, Baghal A, Wongsurawat T, Jenjaroenpun P, Powell T, Al-Shukri S, et al. Chest imaging representing a COVID-19 positive rural US population. Scientific data. 2020;7(1):1–6.
16. Consortium NCICPTA. Radiology Data from the Clinical Proteomic Tumor Analysis Consortium Head and Neck Squamous Cell Carcinoma [CPTAC-HNSCC] Collection. The Cancer Imaging Archive. 2018.
17. Tatum JL, Kalen JD, Ileva LV, Riffle LA, Keita S, Patel N, et al. Imaging characterization of a metastatic patient derived model of adenocarcinoma colon: PDMR-997537-175-T [Data set]. The Cancer Imaging Archive. 2020.
18. Grossberg AJ, Mohamed AS, Elhalawani H, Bennett WC, Smith KE, Nolan TS, et al. Imaging and clinical data archive for head and neck squamous cell carcinoma patients treated with radiotherapy. Scientific data. 2018;5:180173.
19. Elhalawani H, Mohamed AS, White AL, Zafereo J, Wong AJ, Berends JE, et al. Matched computed tomography segmentation and demographic data for oropharyngeal cancer radiomics challenges. Scientific data. 2017;4:170077.
20. Jaggi A, Mattonen SA, McNitt-Gray M, Napel S. Stanford DRO Toolkit: digital reference objects for standardization of radiomic features. Tomography. 2020;6(2):111.
21. Prah M, Stufflebeam S, Paulson E, Kalpathy-Cramer J, Gerstner E, Batchelor T, et al. Repeatability of standardized and normalized relative CBV in patients with newly diagnosed glioblastoma. American Journal of Neuroradiology. 2015;36(9):1654–1661
22. Consortium NCICPTA. Radiology Data from the Clinical Proteomic Tumor Analysis Consortium Glioblastoma Multiforme [CPTAC-GBM] collection [Data set]. The Cancer Imaging Archive. 2018.
23. Consortium NCICPTA. Radiology Data from the Clinical Proteomic Tumor Analysis Consortium Sarcomas [CPTAC-SAR] collection [Data set]. The Cancer Imaging Archive. 2018.
24. Consortium NCICPTA. Radiology Data from the Clinical Proteomic Tumor Analysis Consortium Uterine Corpus Endometrial Carcinoma [CPTAC-UCEC] Collection [Data set]. The Cancer Imaging Archive. 2018.
25. Kwan JYY, Su J, Huang SH, Ghoraie LS, Xu W, Chan B, et al. Radiomic biomarkers to refine risk models for distant metastasis in HPV-related oropharyngeal carcinoma. International Journal of Radiation Oncology* Biology* Physics. 2018;102(4):1107–1116
26. Li X, Abramson RG, Arlinghaus LR, Kang H, Chakravarthy AB, Abramson VG, et al. Multiparametric magnetic resonance imaging for predicting pathological response after the first cycle of neoadjuvant chemotherapy in breast cancer. Investigative radiology. 2015;50(4):195–204.
27. Rusu M, Rajiah P, Gilkeson R, Yang M, Donatelli C, Thawani R, et al. Co-registration of pre-operative CT with ex vivo surgically excised ground glass nodules to define spatial extent of invasive adenocarcinoma on in vivo imaging: a proof-of-concept study. European radiology. 2017;27(10):4209–4217.
28. Kalendralis P, Shi Z, Traverso A, Choudhury A, Sloep M, Zhovannik I, et al. FAIR-compliant clinical, radiomics and DICOM metadata of RIDER, Interobserver, Lung1 and Head-Neck1 TCIA collections. Medical Physics. 2020.
29. Schmainda K, Prah M, Connelly J, Rand S. Glioma DSC-MRI perfusion data with standard imaging and ROIs. The Cancer Imaging Archive http://doi org/107937 K. 2016;9.
30. Mackin D, Fave X, Zhang L, Fried D, Yang J, Taylor B, et al. Court L. Data from Credence Cartridge Radiomics Phantom CT Scans The Cancer Imaging Archive. 2017.
31. Yang J, Veeraraghavan H, Armato III SG, Farahani K, Kirby JS, Kalpathy-Kramer J, et al. Autosegmentation for thoracic radiation treatment planning: A grand challenge at AAPM 2017. Medical physics. 2018;45(10):4568–4581.
32. Patnana M, Patel S, Tsao A. Anti-PD-1 Immunotherapy Melanoma Dataset [Data set]. The Cancer Imaging Archive. 2019.
33. Erickson BJ, Mutch D, Lippmann L, Jarosz R. Radiology Data from The Cancer Genome Atlas Uterine Corpus Endometrial Carcinoma (TCGA-UCEC) collection. The Cancer Imaging Archive. 2016.
34. Zuley ML, Jarosz R, Kirk S, Lee Y, Colen R, Garcia K, et al. Radiology Data from The Cancer Genome Atlas Head-Neck Squamous Cell Carcinoma [TCGA-HNSC] collection. The Cancer Imaging Archive. 2016.
35. Bejarano T, De Ornelas-Couto M, Mihaylov IB. Longitudinal fan-beam computed tomography dataset for head-and-neck squamous cell carcinoma patients. Medical physics. 2019;46(5):2526–2537.
36. Ger RB, Yang J, Ding Y, Jacobsen MC, Cardenas CE, Fuller CD, et al. Synthetic head and neck and phantom images for determining deformable image registration accuracy in magnetic resonance imaging. Medical physics. 2018;45(9):4315–4321.
37. Vallieres M, Kay-Rivest E, Perrin LJ, Liem X, Furstoss C, Aerts HJ, et al. Radiomics strategies for risk assessment of tumour failure in head-and-neck cancer. Scientific reports. 2017;7(1):1–14.
38. Akkus Z, Ali I, Sedlář J, Agrawal JP, Parney IF, Giannini C, et al. Predicting deletion of chromosomal arms 1p/19q in low-grade gliomas from MR images using machine intelligence. Journal of digital imaging. 2017;30(4):469–476.
39. Lee RS, Gimenez F, Hoogi A, Miyake KK, Gorovoy M, Rubin DL. A curated mammography data set for use in computer-aided detection and diagnosis research. Scientific data. 2017;4:170177.
40. Gavrielides MA, Kinnard LM, Myers KJ, Peregoy J, Pritchard WF, Zeng R, et al. A resource for the assessment of lung nodule size estimation methods: database of thoracic CT scans of an anthropomorphic phantom. Optics express. 2010;18(14):15244.
41. Kalpathy-Cramer J, Napel S, Goldgof D, Zhao B. QIN multi-site collection of Lung CT data with nodule segmentations. Cancer Imaging Arch. 2015;10:K9.
42. Jansen S, Van Dyke T. TCIA Mouse-Astrocytoma Collection. The Cancer Imaging Archive. 2015.
43. Albertina B, Watson M, Holback C, Jarosz R, Kirk S, Lee Y, et al. Radiology data from the cancer genome atlas lung adenocarcinoma [tcga-luad] collection. The Cancer Imaging Archive. 2016.
44. Erickson B, Kirk S, Lee Y, Bathe O, Kearns M, Gerdes C, et al. Radiology Data from The Cancer Genome Atlas Liver Hepatocellular Carcinoma [TCGA-LIHC] collectionThe. Cancer Imaging Archive. 2016.
45. Puchalski RB, Shah N, Miller J, Dalley R, Nomura SR, Yoon JG, et al. An anatomic transcriptional atlas of human glioblastoma. Science. 2018;360(6389):660–663.
46. Singanamalli A, Rusu M, Sparks RE, Shih NN, Ziober A, Wang LP, et al. Identifying in vivo DCE MRI markers associated with microvessel architecture and gleason grades of prostate cancer. Journal of Magnetic Resonance Imaging. 2016;43(1):149–158.
47. Toth RJ, Shih N, Tomaszewski JE, Feldman MD, Kutter O, Yu DN, et al. Histostitcher: An informatics software platform for reconstructing whole-mount prostate histology using the extensible imaging platform framework. Journal of Pathology Informatics. 2014;5.
48. Xiao G, Bloch BN, Chappelow J, Genega EM, Rofsky NM, Lenkinski RE, et al. Determining histologyMRI slice correspondences for defining MRI-based disease signatures of prostate cancer. Computerized Medical Imaging and Graphics. 2011;35(7-8):568–578
49. Chappelow J, Bloch BN, Rofsky N, Genega E, Lenkinski R, DeWolf W, et al. Elastic registration of multimodal prostate MRI and histology via multiattribute combined mutual information. Medical Physics. 2011;38(4):2005–2018.
50. Zuley M, Jarosz R, Drake B, et al. Radiology data from the cancer genome atlas prostate adenocarcinoma [TCGA-PRAD] collection. The Cancer Imaging Archive Available online: http://doi org/107937 K. 2016;9.
51. Newitt D, Hylton N. Single site breast DCE-MRI data and segmentations from patients undergoing neoadjuvant chemotherapy. The Cancer Imaging Archive. 2016;2.
52. Barboriak D. Data From RIDER NEUR MRI. The Cancer Imaging Archive; 2015.
53. Vallières M, Freeman CR, Skamene SR, El Naqa I. A radiomics model from joint FDG-PET and MRI texture features for the prediction of lung metastases in soft-tissue sarcomas of the extremities. Physics in Medicine and Biology. 2015;60(14):5471.
54. Jansen S, Ileva L, Lu L, Van Dyke T. TCIA Mouse-Mammary Collection. The Cancer Imaging Archive. 2015
55. Kirk S, Lee Y, Roche C, Bonaccio E, Filippini J, Jarosz R. Radiology data from the Cancer Genome Atlas Thyroid Cancer [TCGA-THCA] collection. Cancer Imaging Archive doi. 2016;10:K9.
56. Roche C, Bonaccio E, Filippini J. cited 2019 18/01/2019. Radiology data from The Cancer Genome Atlas Sarcoma collection The Cancer Imaging Archive 2016. 2016.
57. Grove O, Berglund AE, Schabath MB, Aerts HJ, Dekker A, Wang H, et al. Quantitative computed tomographic descriptors associate tumor shape complexity and intratumor heterogeneity with prognosis in lung adenocarcinoma. PloS one. 2015;10(3):e0118261
58. Lucchesi F, Aredes N. Radiology data from The Cancer Genome Atlas Cervical Squamous Cell Carcinoma and Endocervical Adenocarcinoma (TCGA-CESC) collection. The Cancer Imaging Archive. The Cancer Imaging Archive; 2016.
59. Holback C, Jarosz R, Prior F, Mutch DG, Bhosale P, Garcia K, et al.. Radiology Data from The Cancer Genome Atlas Ovarian Cancer [TCGA-OV] collection. The Cancer Imaging Archive; 2016.
60. Pedano N, Flanders AE, Scarpace L, Mikkelsen T, Eschbacher J, Hermes B, et al. Radiology data from the cancer genome atlas low grade glioma [TCGA-LGG] collection. The Cancer Imaging Archive. 2016;2.
61. Beichel RR, Ulrich EJ, Bauer C, Byrd DW, Muzi JP, Muzi M, et al. Data From QIN PET Phantom. The Cancer Imaging Archive. 2015.
62. Huang W, Li X, Chen Y, Li X, Chang MC, Oborski MJ, et al. Variations of dynamic contrast-enhanced magnetic resonance imaging in evaluation of breast cancer therapy response: a multicenter data analysis challenge. Translational oncology. 2014;7(1):153.
63. Zhao B. Data From Lung Phantom. The Cancer Imaging Archive. 2015.
64. Linehan MW, Gautam R, Sadow CA, Levine S. Radiology Data from The Cancer Genome Atlas Kidney Chromophobe [TCGA-KICH] collection. The Cancer Imaging Archive. 2016.
65. L S, T M, Cha and RS, S T, D G, JH S, et al. Radiology Data from The Cancer Genome Atlas Glioblastoma Multiforme [TCGA-GBM] collection. The Cancer Imaging Archive. 2016.
66. Armato III SG, Drukker K, Li F, Hadjiiski L, Tourassi GD, Engelmann RM, et al. LUNGx Challenge for computerized lung nodule classification. Journal of Medical Imaging. 2016;3(4).
67. Litjens G, Futterer J, Huisman H. Data from prostate-3t: the cancer imaging archive; 2015.
68. Bloch BN, Jain A, Jaffe CC. Data From PROSTATE-DIAGNOSIS. The Cancer Imaging Archive Available online: http://doi org/107937 K. 2015;9.
69. Muzi P, Wanner M, Kinahan P. Data From RIDER PHANTOM PET-CT. The Cancer Imaging Archive. 2015
70. Zhao B, James LP, Moskowitz CS, Guo P, Ginsberg MS, Lefkowitz RA, et al. Evaluating variability in tumor measurements from same-day repeat CT scans of patients with non–small cell lung cancer. Radiology. 2009;252(1):263–272.
71. Jackson EF, Barboriak DP, Bidaut LM, Meyer CR. Magnetic resonance assessment of response to therapy: tumor change measurement, truth data and error sources. Translational Oncology. 2009;2(4):211.
72. Meyer CR, Chenevert TL, Galbán CJ, Johnson TD, Hamstra DA, Rehemtulla A, et al.. Data from RIDERBreast-MRI. The cancer imaging archive; 2015.
73. Roth HR, Lu L, Farag A, Shin HC, Liu J, Turkbey EB, et al. Deeporgan: Multi-level deep convolutional networks for automated pancreas segmentation. In: International conference on medical image computing and computer-assisted intervention. Springer; 2015. p. 556–564.
74. Roth HR, Lu L, Seff A, Cherry KM, Hoffman J, Wang S, et al. A new 2.5 D representation for lymph node detection using random sets of deep convolutional neural network observations. In: International conference on medical image computing and computer-assisted intervention. Springer; 2014. p. 520–527.
75. Lucchesi FR, Aredes ND. Radiology Data from The Cancer Genome Atlas Esophageal Carcinoma [TCGAESCA] collection. The Cancer Imaging Archive. 2016.
76. Lucchesi F, Aredes N. Radiology Data from The Cancer Genome Atlas Stomach Adenocarcinoma [TCGASTAD] collection, 2016. The Cancer Imaging Archive;10:K9.
77. Armato III SG, McLennan G, Bidaut L, McNitt-Gray MF, Meyer CR, Reeves AP, et al. The lung image database consortium (LIDC) and image database resource initiative (IDRI): a completed reference database of lung nodules on CT scans. Medical physics.2011;38(2):915–931.
78. Fenimore C, McNitt-Gray MF, Clunie D, Gavrielides MA, Petrick N, Samei E, et al. Data from QIBA CT-1C. The Cancer Imaging Archive. 2016.
79. P C, B T, P P, M M, B W. Data From PROSTATE-MRI. The Cancer Imaging Archive. 2016.
80. Wang X, Peng Y, Lu L, Lu Z, Bagheri M, Summers RM. Chestx-ray8: Hospital-scale chest x-ray database and benchmarks on weakly-supervised classification and localization of common thorax diseases. In: Proceedings of the IEEE conference on computervision and pattern recognition; 2017. p. 2097–2106.
81. Bien N, Rajpurkar P, Ball RL, Irvin J, Park A, Jones E, et al. Deep-learning-assisted diagnosis for knee magnetic resonance imaging: development and retrospective validation of MRNet. PLoS medicine. 2018;15(11):e1002699.
82. Consortium NCICPTA. Radiology Data from the Clinical Proteomic Tumor Analysis Consortium Clear Cell Renal Cell Carcinoma [CPTAC-CCRCC] collection [Data set]. The Cancer Imaging Archive. 2018.
83. Heller N, Isensee F, Maier-Hein KH, Hou X, Xie C, Li F, et al. The state of the art in kidney and kidney tumor segmentation in contrast-enhanced ct imaging: Results of the kits19 challenge. Medical Image Analysis. 2019;67:101821.
84. Consortium NCICPTA. Radiology Data from the Clinical Proteomic Tumor Analysis Consortium Lung Squamous Cell Carcinoma [CPTAC-LSCC] Collection [Data set]. The Cancer Imaging Archive. 2018.
85. Moen TR, Chen B, Holmes III DR, Duan X, Yu Z, Yu L, et al. Low Dose CT Image and Projection Datas et. Medical Physics. 2020.
86. Consortium NCICPTA. Radiology Data from the Clinical Proteomic Tumor Analysis Consortium Lung Adenocarcinoma [CPTAC-LUAD] collection [Data set]. The Cancer Imaging Archive. 2018.
87. Consortium NCICPTA. Radiology Data from the Clinical Proteomic Tumor Analysis Consortium Pancreatic Ductal Adenocarcinoma [CPTAC-PDA] Collection [Data set].. The Cancer Imaging Archive. 2018.
88. Yorke A, Sala I, Solis D, Guerrero T. A Statistically Characterized Reference Data Set for Image Registration of Pelvis Using Combinatorial Affine Registration Optimization. In: MEDICAL PHYSICS. vol. 46. WILEY 111 RIVER ST, HOBOKEN 07030-5774, NJUSA; 2019. p. E340–E340.
89. Madhavi P, Patel S, Tsao AS. Data from Anti-PD-1 Immunotherapy Lung [Data set]. The Cancer Imaging Archive. 2019.
90. Hylton NM, Gatsonis CA, Rosen MA, Lehman CD, Newitt DC, Partridge SC, et al. Neoadjuvant chemotherapy for breast cancer: functional tumor volume by MR imaging predicts recurrence-free survival—results from the ACRIN 6657/CALGB 150007 I-SPY 1 TRIAL. Radiology. 2016;279(1):44–55.
91. Fedorov A, Clunie D, Ulrich E, Bauer C, Wahle A, Brown B, et al. DICOM for quantitative imaging biomarker development: a standards based approach to sharing clinical data and structured PET/CT analysis results in head and neck cancer research. PeerJ. 2016;4:e2057.
92. Kirk S, Lee Y, Sadow CA, Levine S. Radiology Data from The Cancer Genome Atlas Rectum Adenocarcinoma [TCGA-READ] collection. The Cancer Imaging Archive. 2016.
93. Kurdziel KA, Shih JH, Apolo AB, Lindenberg L, Mena E, McKinney YY, et al. The kinetics and reproducibility of 18F-sodium fluoride for oncology using current PET camera technology. Journal of Nuclear Medicine. 2012;53(8):1175–1184.
94. Scarpace L, Flanders AE, Jain R, Mikkelsen T, Andrews DW. Data from REMBRANDT. The Cancer Imaging Archive. 2015;10:K9.
